# Simultaneous determination of free methamphetamine, pethidine, ketamine and tramadol in urine by dispersive liquid–liquid microextraction combined with GC–MS

**DOI:** 10.1080/20961790.2017.1377386

**Published:** 2017-09-27

**Authors:** Fangmin Xu, Lingyun Liu

**Affiliations:** Institute of Forensic Science, Public Security Bureau of Jiangyin, Jiangsu, China

**Keywords:** Forensic science, forensic toxicology, dispersive liquid–liquid microextraction, gas chromatography mass spectrometry, urine, drug abuse, sample pretreatment

## Abstract

A simple and rapid dispersive liquid–liquid microextraction (DLLME) technique coupled with gas chromatography–ion trap mass spectrometry (GC–MS) was developed for the extraction and analysis of methamphetamine (MA), pethidine (PD), ketamine (KT) and tramadol (TD) from human urine. In this study, different parameters affecting the extraction process such as the type and volume of extraction solvent, type and volume of disperser solvent, extraction time and pH value and salt effect were studied and optimized. Under optimized conditions, the enrichment factor ranged from 185 to 226 and the average recovery ranged from 80.45% to 95.55%. The linear range was 10.0–1000.0 µg/L, the limit of detection and quantitation were in the range 0.43–1.96 µg/L and 1.44–6.53 µg/L, respectively. The relative standard deviations were in the range 1.98%–3.90% (*n* = 7). The obtained results show that DLLME combined with GC–MS is a fast and simple method for the determination of MA, PD, KT and TD in human urine.

## Introduction

Around the world, drug abuse is increasing rapidly, especially among young people. Drug abuse not only harms them psychologically and physically, but also causes serious social problems. Methamphetamine (MA) is a strong central nervous system stimulant, which is the most popular abused drug due to its low price and wide availability [1,2]. Ketamine (KT) is a medication used as anesthetic in both animals and humans. Because of its hallucinogenic properties, it is a popular drug of abuse among young people [2]. Pethidine (PD) and tramadol (TD) are both opioid pain medication drugs, which are used to treat moderate to severe pain. Sometimes, PD and TD also are abused because of the lower risk of addiction. Therefore, it is important to establish a simple, direct and sensitive preconcentration method for the determination of the four drugs [3,4]. Conventional extraction methods such as liquid–liquid extraction (LLE) [5,6] and solid-phase extraction (SPE) [7] require large volumes of organic solvents and are time-consuming. To deal with these disadvantages, solid-phase microextraction (SPME) [2,8] has been developed. SPME uses no extraction solvent, but it is expensive, and its fibre is fragile and has limited lifetime and sample carry-over can be another problem. In recent years, a few new preconcentration technologies have been introduced, such as hollow-fibre-protected liquid-phase microextraction [9], liquid membrane extraction (LME) [10], molecularly imprinted polymer solid-phase microextraction [11,12]. All of these techniques have their own advantages; however, they can also be relatively expensive and require long extraction times. So, a novel microextraction technique, dispersive liquid–liquid microextraction (DLLME) [13] had been developed. DLLME is simple, rapid and affords high enrichment factor. This is due to the large contact surface area of the extraction solvent [14], which has many advantages including simplicity of operation, rapidity, low cost, high recovery and enrichment factors, and they have been widely used in analytical chemistry [15–17].

In this study, we report a simple and rapid DLLME method coupled with gas chromatography mass spectrometry (GC–MS) for the analysis of MA, KT, PD and TD in human urine. The factors which affect DLLME extraction were investigated and operating conditions were optimized. Under optimized conditions, four free drugs were analyzed simultaneously in human urine.

## Material and methods

### Reagents and standards

MA, KT, PD and TD were purchased from the National Institute for the Control of Pharmaceutical and Biological Products (Beijing, China) and the purity was above 98%. Tetrachloroethylene (C_2_Cl_4,_ purity, 99%), carbon tetrachloride (CCl_4_, purity, 99.5%) and chlorobenzene (C_6_H_5_Cl, purity, 99%) were obtained from J&K Chemical Ltd, Shanghai. These solvents were used as extraction solvents. Acetone (C_3_H_6_O, purity, 99.5%), acetonitrile (C_2_H_3_N, purity, 99.9%), methanol (CH_4_O, purity, 99.9%) and ethanol (C_2_H_6_O, purity, 99.9%) as disperser solvent were obtained from Sigma. Sodium hydroxide (NaOH), hydrochloric acid (HCl), sodium bicarbonate (NaHCO_3_), sodium carbonate (Na_2_CO_3_) and sodium chloride (NaCl) were the analytical reagents and were purchased from Shanghai Chemical Reagent Co. Ltd.

### Instrumentation

Analysis of the four drugs was performed on a Varian Saturn 2200 GC/MS system. Ultra pure helium (99.99%) was passed through a water trap and oxygen trap before its use as the carrier gas. The GC CP-3800 (Varian) was fitted with a VF-5 column (30 m × 0.25 mm, 0.25 µm) obtained from Agilent Technologies. Helium was used as the carrier gas at a flowrate of 1.0 mL/min. The oven temperature programme employed for the separation of drugs was as follows: 80 °C for 1 min; 20 °C/min to 280 °C, held for 9 min and the CP-1177 split/splitless injector was made in the splitless mode, and the injector temperature was 280 °C. The mass detector was used in the electron impact (EI, 70 eV) mode and scanned over the range *m*/*z* 50–550 to confirm the retention times of the analytes. The trap and transfer line temperatures were 150 °C and 230 °C, respectively. A Sorvall TDL-80-2B (Shanghai Anting Scientific Instrument Factory, Shanghai, China) was used for centrifuging.

### Preparation of standard solutions

MA, KT, PD and TD (10.0 mg for each) was dissolved respectively in 10 mL methanol to obtain a standard stock solution with a concentration of 1.0 mg/mL and stored at −18 °C. Each fresh 0.1 mg/mL standard solution containing the four drugs respectively was prepared in methanol every week and stored at −18 °C. The working solutions were prepared daily by using standard solutions with suitable dilutions.

Drug-free human urine samples (1.0 L) were obtained from two volunteers (laboratory staff) and were stored at −18 °C. The urine samples were thawed at room temperature and centrifuged before analysis. The blank urine samples were used for the validation of the analytical method.

### DLLME procedure

Blank urine sample (5.0 mL) was placed in a 15.0 mL glass test tube with conical bottom with each drug at suitable concentration. The pH value was adjusted to 10.0 with 5% NaOH, 1 mol/L HCl and Na_2_CO_3_–NaHCO_3_ buffer solution. The disperser solvent, containing the extraction solvent, was rapidly injected into the sample solution with a syringe. A cloudy solution (urine, disperser solvent and extraction solvent) was formed in the test tube; the cloudy state was stable for a long time. The mixture was let to stand for a few minutes, and then centrifuged at 4000 rpm, causing the dispersed droplets of the extraction phase to settle at the bottom of the conical test tube. The 1.0 µL of sediment extraction phase was collected using a 10.0 µL micro-syringe and injected into the GC–MS system. The volume of the sediment phase was determined using a 100.0 µL micro-syringe.

### Calculation of enrichment factor and extraction recovery

The enrichment factor (EF) is defined as the ratio between the analyte concentration in the sedimented phase (*C*_sed_) and the initial concentration of analyte (*C*_0_) within the sample:

$$\text{EF}=\frac{C_{\text{sed}}}{C_{0}}$$

The concentration of analyte was obtained from the calibration graph of direct injection of standard solution at the suitable range.

The extraction recovery (ER) is defined as the percentage of the total analyte amount (*n*_0_) which was extracted to the sedimented phase (*n*_sed_):

$$\text{ER}\;(\%)=\frac{n_{\text{sed}}}{n_{0}}\times 100=\frac{C_{\text{sed}}\times V_{\text{sed}}}{C_{0}\times V_{0}}\times 100=\text{EF}\times \frac{V_{\text{sed}}}{V_{0}}\times 100$$

in which *V*_sed_ and *V*_0_ are the volumes of sedimented phase and sample solution, respectively.

## Results and discussion

In order to obtain the optimization extraction performance, different factors, such as selection of suitable extraction solvent, selection of suitable disperser solvent, volume of extraction solvent, volume of disperser solvent, pH value, extraction time and ionic strength, were studied.

### Selection of extraction solvent and disperser solvent

**Figure 1.**
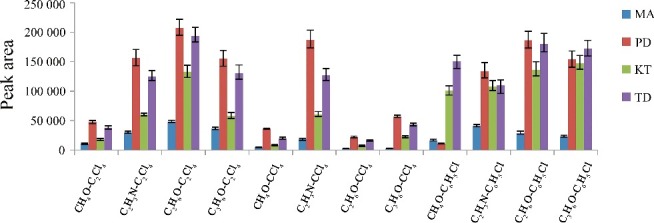
Efficiency of different extraction solvent and disperser solvent evaluated for extraction of the four drugs by DLLME. Extraction conditions: sample volume, 5.00 mL; extraction solvent volume, 20 μL; disperser solvent volume, 0.5 mL; room temperature; concentration of each drug, 0.1 μg/mL.

The selection of an appropriate solvent is more important for the DLLME process. Extraction solvents are selected on the basis of higher density rather than urine, extraction capability of interested compounds and good gas chromatography behaviour. In this study, C_2_Cl_4_, CCl_4_ and C_6_H_5_Cl were compared in the extraction of the four drugs. Dispersive solvents should be miscible solvents with both aqueous samples and extraction solvents to help the analytes transfer from aqueous phase into organic phase. C_3_H_6_O, C_2_H_3_N, CH_4_O and C_2_H_6_O were studied as dispersive solvents. Thus, a series of solvents were compared for the extraction of the four drugs, and were evaluated for extraction efficiency using the following model: 5.00 mL of blank urine sample with each drug at a concentration of 100 μg/L, 0.50 mL of dispersive solvent and 20.0 μL of extraction solvent were used. The extraction efficiency was evaluated by comparison of the peak area of each analyte. The peak area of each analyte is shown in Figure 1.

The results revealed that the series of C_2_H_6_O (dispersive solvent)–C_2_Cl_4_ (extraction solvent) and C_2_H_6_O (dispersive solvent)–C_6_H_5_Cl (extraction solvent) have the highest extraction efficiency in comparison with the other series. However, the series of C_2_H_6_–C_6_H_5_Cl has higher background interference. Thereby, the series of C_2_Cl_4_–C_2_H_6_O was selected as the extraction solvent and dispersive solvent, respectively.

### Optimization of extraction solvent volume

**Figure 2.**
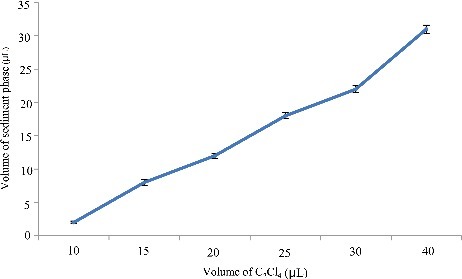
Efficiency of the volume of C_2_Cl_4_ evaluated for extraction of the four drugs by DLLME. Extraction conditions: sample volume, 5.00 mL; extraction solvent volume, 5.0, 10.0, 15.0, 20.0, 25.0, 30.0 and 40.0 µL; disperser solvent volume, 0.5 mL; room temperature; concentration of each drug, 0.1 μg/mL.

**Figure 3.**
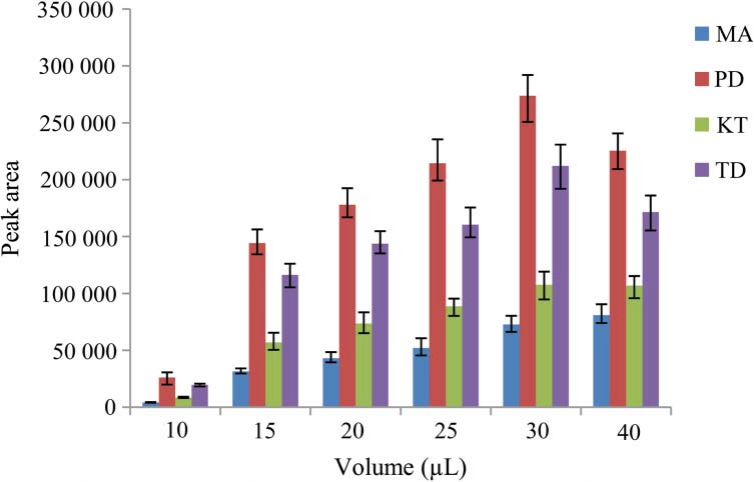
Efficiency of the volume of C_2_Cl_4_ on the volume of sediment phase in DLLME. Extraction conditions: sample volume, 5.00 mL; extraction solvent volume, 5, 10.0, 15.0, 20.0, 25.0, 30.0 and 40.0 µL; disperser solvent volume,0.5 mL; room temperature; concentration of each drug, 0.1 μg/mL.

To study the effect of extraction solvent volume, disperser solvent containing different volumes of C_2_Cl_4_ was subjected to exactly the same DLLME procedure. The experimental conditions were fixed and included the use of a constant volume of C_2_H_6_O (0.5 mL) containing different volumes of C_2_Cl_4_ (5.0, 10.0, 15.0, 20.0, 25.0, 30.0 and 40.0 µL). Figures 2 and 3 show the curve of the volume of sediment phase and the histogram of peak area versus the volume of extraction solvent (C_2_Cl_4_), respectively. According to Figure 2, by increasing the volume of C_2_Cl_4_ from 5.0 to 40.0 µL, the volume of the sediment phase increases from 0.0 to 31.0 µL. For 5.0 µL, no sediment phases were obtained after centrifugation, so 5.0 µL was rejected. Regarding Figure 3, by increasing the volume of C_2_Cl_4_, the peak areas increased due to increase in the volume of organic phase collected after extraction which in turn leads to increase in analytes concentrations in the organic phase. However, when the volume of C_2_Cl_4_ was set to 30.0 µL, the peak area for most analytes reaches the maximum, which indicates the quantitative extraction and high distribution coefficients of the four drugs in this condition. Thereby, good sensitivity was achieved by using 30.0 µL volume of C_2_Cl_4_.

### Optimization of disperser solvent volume

**Figure 4.**
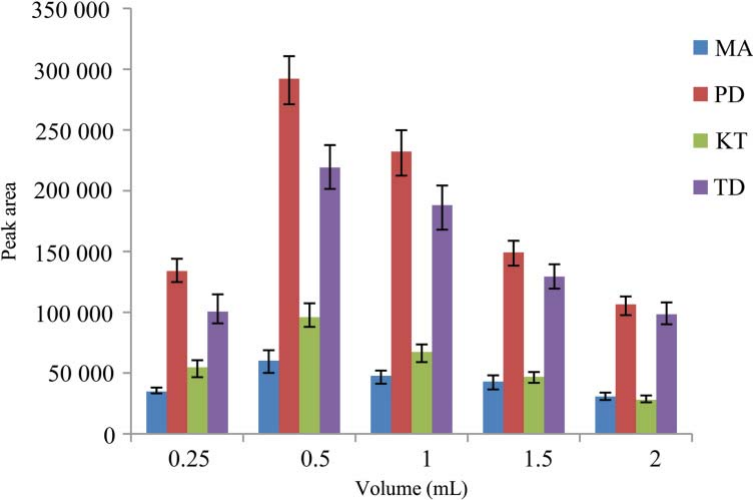
Efficiency of the volume of C_2_H_6_O evaluated for extraction of the four drugs by DLLME. Extraction conditions: sample volume, 5.00 mL; extraction solvent volume, 30.0 µL; disperser solvent volume, 0.25, 0.50, 1.00, 1.50 and 2.00 mL; room temperature; concentration of each drug, 0.1 μg/mL.

In this work, changing the volume of the disperser solvent might be effective on the extraction efficiency. Hence, to obtain the optimum volume of the disperser solvent, various volumes of C_2_H_6_O (0.25, 0.50, 1.00, 1.50, 2.00 mL) containing 30.0 µL extraction solvent (C_2_Cl_4_) were studied. The results are shown in Figure 4. According to the histogram, the extraction efficiency of the analytes was increased and then decreased by increasing the volume of C_2_H_6_O. It seems, at a low volume of C_2_H_6_O, cloudy state is not formed well, thereby, the extraction recovery decreases. At high volume of C_2_H_6_O, the solubility of the four drugs in urine increases, therefore, the extraction efficiency decreases. A 0.5 mL of C_2_H_6_O was chosen as the optimum volume.

### Effect of extraction time

Time is the most important factor in the mass transfer of analytes from the sample solution to the extraction solvent; therefore, this factor is evaluated in the paper. In DLLME extraction, time is defined as the time interval between injecting the mixture of the disperser solvent and the extraction solvent and starting to centrifuge. Under constant experimental conditions, the effect of time was set at 0.5, 1.0, 2.0, 4.0 and 10.0 min, respectively.

**Figure 5.**
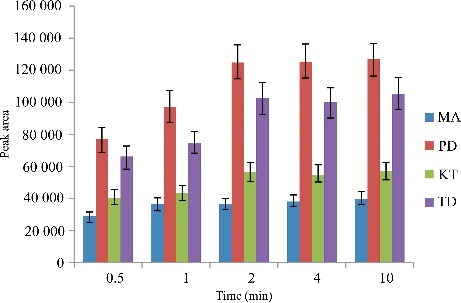
Efficiency of the extraction time evaluated for extraction of the four drugs by DLLME. Extraction conditions: sample volume, 5.00 mL; extraction solvent volume, 30.0 µL; disperser solvent volume, 0.5 mL; room temperature; concentration of each drug, 0.1 μg/mL.

Figure 5 shows the peak areas of the four drugs versus the extraction time. Because of the infinitely large surface area between the extraction solvent and the aqueous phase after the formation of cloudy solution, the mass transfer of analytes is so fast that the extraction equilibrium can be achieved in a short time. According to the graph, the peak area of the four drugs increases so fast, which reaches the maximum and then levels off. Therefore, not less than 2.0 min was chosen as the optimum time.

### Effect of pH value

**Figure 6.**
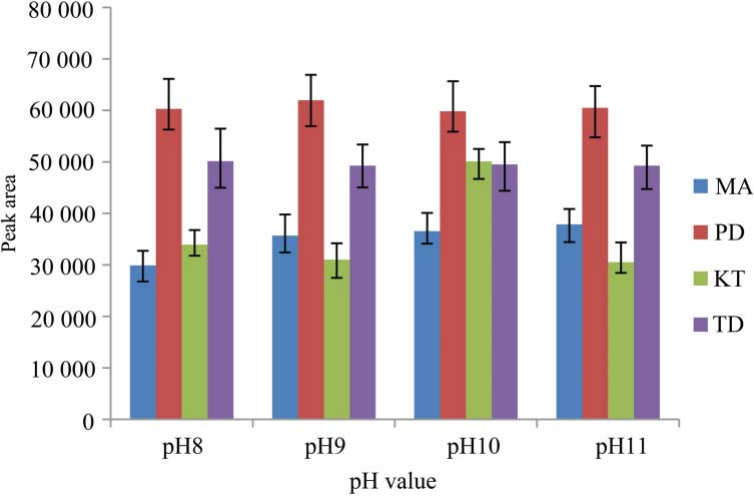
Efficiency of the sample pH value evaluated for extraction of the four drugs by DLLME. Extraction conditions: sample volume, 5.00 mL; extraction solvent volume, 30.0 µL; disperser solvent volume, 0.5 mL; room temperature; concentration of each drug, 0.1 μg/mL.

The pH value of the sample is also an important factor in the preconcentration techniques, which determines the extraction efficiency. Therefore, the effect of sample pH value also needs to be investigated. In this paper, the pH value of the sample was adjusted from 8 to 11 via adding 5% NaOH, 1 mol/L HCl and Na_2_CO_3_–NaHCO_3_ buffer solution in the samples. The results are shown in Figure 6.

According to Figure 6, the peak areas of PD and TD were basically unchanged in the pH range 8–11. The peak area of KT increased with the increase of pH value in the pH range 8–10, and decreased in the pH range 10–11. The peak area of MA increased with the increase of pH value in the pH range 8–9, and which reached a new balance from pH 9 to pH 11. In summary, the pH value of the sample was adjusted as 10 in the experiment.

### Effect of ionic strength

**Figure 7.**
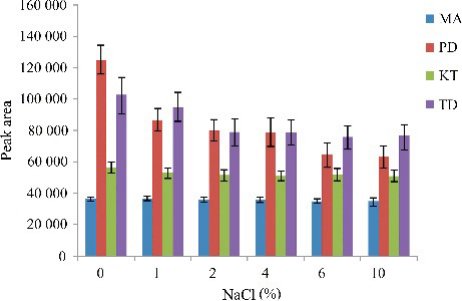
Efficiency of salt evaluated for extraction of the four drugs by DLLME. Extraction conditions: sample volume, 5.00 mL, extraction solvent volume, 30.0 µL; disperser solvent volume, 0.5 mL; room temperature; concentration of each drug, 0.1 μg/mL.

Ionic strength effect is an important factor in the DLLME process, which was evaluated by adding NaCl. The addition of salt can reduce the solubility of target analytes in water while enhancing their transfer into organic solvents. In this work, NaCl was added into the aqueous phase in the range of 0%–10%. As shown in Figure 7, with increase in the concentration of NaCl from 0% to 10%, the extraction efficiencies of the analytes slightly decreased. This could be due to the increased ionic strength, which reduces the diffusion rate of the analytes into the extracting solvent. Thus, no salt addition was used in this study.

### Quantitative analysis

**Figure 8.**
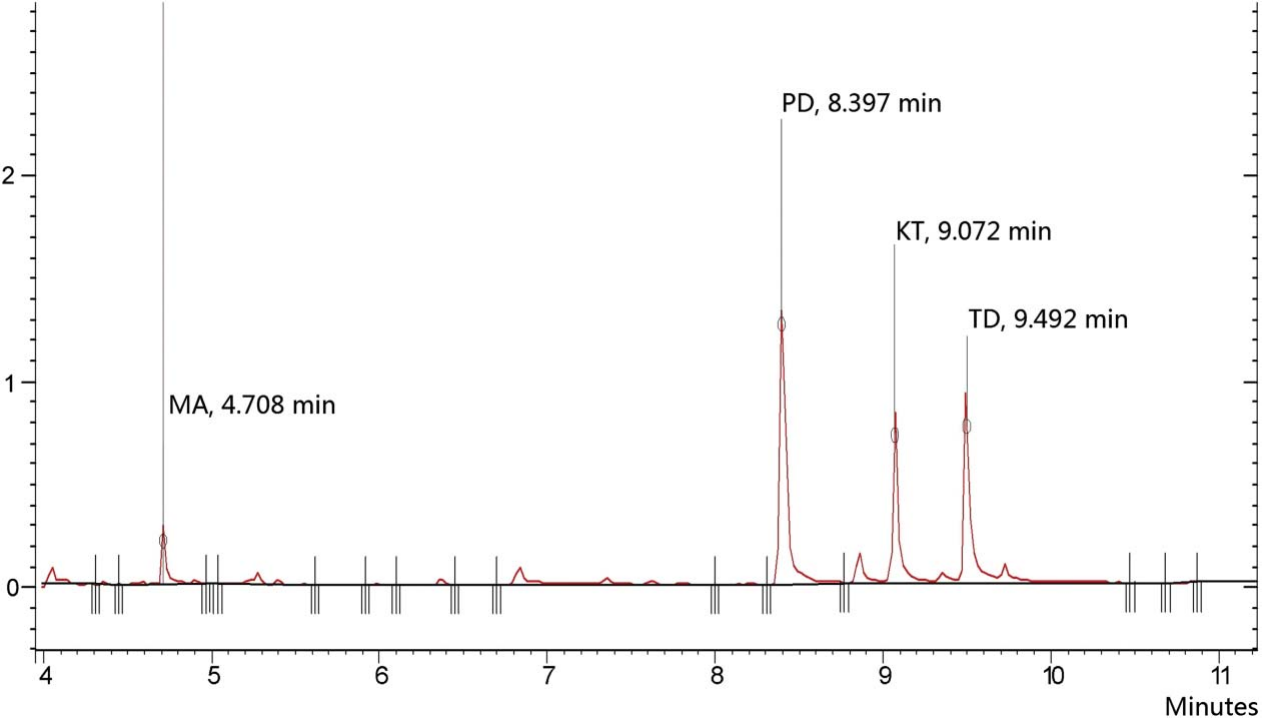
Total ion chromatogram of the four drugs. Extraction conditions: sample volume, 5.00 mL; extraction solvent volume, 30.0 µL; disperser solvent volume, 0.5 mL; room temperature; concentration of each drug, 0.1 μg/mL.

Analytical characteristics of the method were evaluated in the determination of the four drugs according to the DLLME procedure under the optimized conditions. The total ion chromatogram of the four drugs is shown in Figure 8.

Some analytical features such as enrichment factor, linear range, correlation coefficient, limit of detection (LOD), limit of quantification and repeatability were investigated. Table 1 shows the quantification and diagnostic ions of the four drugs. Table 2 summarizes the analytical characteristics of the optimized method. Linearity of calibration curve was observed at 10, 20, 50, 100, 200, 500 and 1000 µg/L for the analytes. Coefficient of correlation (*r*^2^) ranged from 0.991 to 0.998. The repeatability was studied by extracting the samples containing each drug at 100 µg/L. The relative standard deviations (RSD) were calculated to be in the range of 1.98%–3.90% for seven repeated experiments. The limits of quantification (LOQ), based on signal-to-noise ratio (S/N) of 10 ranged from 1.44 to 6.53 µg/L, and the LOD based on S/N of three ranged from 0.43 to 1.96 µg/L, which is very low by using GC–MS.

**Table 1.** Quantification and diagnostic ions used in GC–MS analysis.

Analyte	Retention time (min)	Molecular mass	Quantification ions (base ions, *m*/*z*)	Diagnostic ion (*m*/*z*)
MA	4.708	149.2	58	91 (17%)
PD	8.397	247.3	71	172 (70%), 70 (50%)
KT	9.072	237.7	180	182 (35%), 209 (25%)
TD	9.492	263.4	58	263 (10%), 135 (7%)

Inside brackets refer to the relative abundance of ions (m/z) for each analyte.

**Table 2.** Quantitative features of the method for the four drugs.

Analyte	Linear range (µg/L)	*r*^2^	RSDs (*n* = 7, %)	LOQs (µg/L)	LODs (µg/L)	EF	Recovery (%)
MA	10.0–1000.0	0.998	3.90	6.53	1.96	226	95.55
PD	10.0–1000.0	0.991	3.78	1.44	0.43	190	82.85
KT	10.0–1000.0	0.996	3.40	3.03	0.91	204	90.63
TD	10.0–1000.0	0.994	1.98	2.57	0.77	185	80.45

### Comparison of DLLME with LLE and SPE

Table 3 shows a comparison of the LLE and SPE with others available in the literature. The linear range, recovery, LOD in the LLE and SPE for extraction abuse drugs from body fluid are shown in Table 3. The comparison of the results shows that DLLME has low LOD, wide linear range and high recovery, and it is very simple, rapid and low cost to use.

**Table 3.** Comparison of the method with other studies.

Method	Analyte	Linear range	Concentration (μg/mL)	Recovery (%)	LOD	Reference
LLE–GC/MS	MA, PD, TD, etc.	10–5000 ng/mL	2.5	63.3–81.1	2 ng/mL	[18]
SPE–GC/MS	MA, PD, TD, etc.	10–5000 ng/mL	2.5	66.8–98.5	2 ng/mL	[18]
SPE–GC/MS	MA, KT, etc.	5–200 ng/mL	0.1	95.4–96.8	2.5 ng/mL	[19]
SPE–GC/MS	MA, KT, TD, etc.	0.2–50 μg/mL	10	30–82	0.06–2 μg/mL	[20]
LLE–GC/MS	MA, PD, TD, KT	–	0.5	63–104	25–50 ng/mL	[21]
SPE–GC/MS	PD	1.25–40 ng/mL	0.02	109 ± 10.9	0.5 ng/mL	[22]
DLLME–GC/MS	MA, PD, TD, KT	10–1000 ng/mL	0.1	80.45–95.55	0.43–1.96 ng/mL	Present work

### Real forensic sample analysis

A young man was arrested because of abusing TD. The urine was obtained from the suspect, and was submitted for detection in our lab. DLLME–GC/MS was used for detection in this case.

Under optimized DLLME procedures, the results showed the existence of TD in the urine, and the concentration was 5.32 μg/mL.

## Conclusion

In this study, a simple, rapid and inexpensive microextraction technique has been coupled to a GC–MS method for the determination of MA, PD, KT and TD in human urine. The optimized conditions of extraction have been obtained. The experimental results reveal that this method provides high extraction efficiency within a short time compared to other techniques, good selectivity and repeatability, low LODs and LOQs and good linearity over the investigated concentration range. Comparison of this method with other extraction methods such as LLE and SPE shows that the DLLME is simple, rapid, highly efficient and inexpensive. Therefore, the DLLME is a powerful tool for the analysis of MA, PD, KT and TD in human urine.
